# Do 5-Alpha Reductase Inhibitors Influence the Features of Suspicious Lesions on Magnetic Resonance Imaging and Targeted Biopsy Results for Prostate Cancer Diagnosis? †

**DOI:** 10.3390/diagnostics14222567

**Published:** 2024-11-15

**Authors:** Ziv Savin, Avishay Shem-Tov Dlugy, Miri Grinbaum, Tomer Mendelson, Karin Lifshitz, Roy Mano, Gal Keren-Paz, Yuval Bar-Yosef, Rina Neeman, Ofer Yossepowitch, Snir Dekalo

**Affiliations:** 1 Department of Urology, Sourasky Medical Center, Tel Aviv-Yafo 6423906, Israeltomer.mend@gmail.com (T.M.); karinlif@gmail.com (K.L.); galkp@tlvmc.gov.il (G.K.-P.);; 2 Tel Aviv School of Medicine, Tel Aviv University, Tel Aviv-Yafo 6997801, Israel; 3 Department of Medical Imaging, Sourasky Medical Center, Tel Aviv-Yafo 6423906, Israel

**Keywords:** 5-alpha reductase inhibitor, magnetic resonance imaging, prostate biopsy, prostate cancer

## Abstract

Background: 5-alpha reductase inhibitors (5-ARIs) change hormonal pathways and reduce prostate size. We evaluated the effects of 5-ARIs on prostatic multiparametric magnetic resonance imaging (mpMRI) suspicious findings and in the identification of prostate cancer using targeted biopsies. Methods: We conducted a retrospective study including 600 consecutive patients who, between 2017 and 2021, underwent combined transperineal fusion biopsies. Primary outcomes were Prostate Imaging Reporting and Data System version 2 (PIRADS v2) scores and the identification of clinically significant prostate cancer from suspicious lesions (targeted CSPC). Outcomes were compared between patients treated with 5-ARIs for a minimum of 6 months and the other patients. Results: Patients treated with 5-ARIs were older (*p* < 0.001) with higher rates of previous prostate biopsies (*p* = 0.004). PIRADS scores were 3, 4, and 5 in 15 (29%), 28 (54%), and 9 (17%) patients among the 5-ARI group and 130 (24%), 308 (56%), and 110 (20%) patients among the others, and the scores were not different between the groups (*p* = 0.69). The targeted CSPC identification rate among 5-ARI patients was 31%, not different compared to the non-5-ARI group (*p* = 1). Rates of targeted CSPC for each PIRADS score were not affected by 5-ARI treatment. The 5-ARI was not associated with neither PIRADS ≥ 4 score nor targeted CSPC on logistic regression analyses (OR = 0.76, 95% CI 0.4–1.4 and OR = 1.02, 95% CI 0.5–1.9, respectively). Conclusions: 5-ARI treatment is not associated with PIRADS score alterations or targeted biopsy results. Patients treated by 5-ARIs with suspicious lesions should not be addressed differently during the mpMRI-related diagnostic process.

## 1. Introduction

Prostate cancer is a major public health problem across the globe. With 1.3 million new cases and 359,000 deaths in 2020, prostate cancer is the second most common cancer and the fifth leading cause of cancer death in men worldwide. It is the leading cause of cancer death in men in 46 countries. Prostate cancer mortality has been decreasing in many countries due to screening, early detection, and improved treatment [1].

Prostate cancer early detection is nowadays focused on targeting men who harbor clinically significant prostate cancer (CSPC). This paradigm shift resulted from the evidence showing that the detection of favorable-risk prostate cancer can be avoided, and that active surveillance may be offered as a management approach for favorable-risk prostate cancer to spare overtreatment of indolent disease [2]. Definitive treatment with surgery or radiation is either delayed if there are signs and/or symptoms of cancer progression or avoided altogether if no progression is detected, mitigating common adverse effects associated with treatment, such as urinary incontinence, urinary irritative symptoms, and erectile or bowel dysfunction [3]. One of the main tools that made this paradigm shift feasible is the introduction and widespread adoption of multiparametric magnetic resonance imaging (mpMRI) for the selection of candidates for prostate biopsy among men suspected of having prostate cancer [4].

5-alpha reductase inhibitors (5-ARIs) induce hormonal effects both systematically and locally in the prostate and are licensed for men with benign prostatic enlargement [5,6] 5α-reductase is an intracellular enzyme that converts testosterone into the more potent dihydrotestosterone (DHT), which has a stronger affinity for androgen receptors in the prostate. By competitively inhibiting 5α-reductase, 5-ARIs prevent this conversion, thereby reducing DHT levels and modulating the genes responsible for cell proliferation, potentially mitigating hyperplasia. 5α-reductase exists in two isoenzymes, type I and type II, with type II accounting for the majority of serum DHT production. 5-ARIs competitively inhibit the activity of the 5α-reductase enzyme, leading to the suppression of dihydrotestosterone (DHT). Finasteride specifically inhibits the type II 5α-reductase isoenzyme, reducing serum DHT levels by approximately 70% from baseline. In contrast, dutasteride, the first dual inhibitor of both type I and type II 5α-reductase isoenzymes, is estimated to lower serum DHT by around 90% [7]. These effects influence the size of the prostate and the interpretation of prostatic-specific antigen (PSA) levels [5]. 5-ARIs also reduce the proportion of men diagnosed with prostate cancer and the rates of disease progression during active surveillance [8,9]. The usage of 5-ARIs has been largely increased due to its long-term benefit in men with prostatic enlargement, improving both symptoms and preventing complications [6].

Multiparametric magnetic resonance imaging (mpMRI) has become an integral part of the diagnostic process for prostate cancer and guidelines recommend performing mpMRI before prostate biopsy. Suspicious prostatic lesions on mpMRI are stratified according to the Prostate Imaging Reporting and Data System version 2 (PIRADS v2), and targeted biopsies are recommended for lesions scored as PIRADS ≥ 3 [10]. The yield of mpMRI is substantial in avoiding the identification of insignificant prostate cancer on the one hand and increasing the rates of significant cancer on the other. The diagnostic pathway including mpMRI-targeted biopsies in the presence of a lesion suggestive of cancer is considered superior to the diagnostic pathway of standard systematic biopsies [4].

Although a few studies have investigated the relation between 5-ARI treatment, mpMRI findings, and biopsy results [11,12,13,14,15], the effects of 5-ARIs on mpMRI suspicious lesions and their histologic results are still unknown. Our goal was to explore the effects of 5-ARIs on mpMRI suspicious lesions and on detection rates of CSPC using mpMRI-targeted biopsies.

## 2. Materials and Methods

### 2.1. Patients

After obtaining Institutional Review Board approval, we conducted a retrospective study that included patients who performed mpMRI of the prostate, had suspicious lesions classified as PIRADS ≥ 3, and underwent mpMRI-targeted transperineal fusion biopsies in our institute between March 2017 and March 2021. All patients in the cohort performed their mpMRI due to elevated PSA, abnormal rectal examination, or active surveillance. The study cohort was stratified according to 5-ARI treatment for a minimum of 6 months from the time the mpMRI was performed. Clinical and demographic characteristics were compared between the groups, including age, family history, PSA, and cT staging.

### 2.2. mpMRI Protocol

The mpMRI scans were performed on a 3T scanner with a 16-channel surface coil. According to PIRADS v.2 guidelines, all scans included T2 Echo sequences on three scan planes, a T1 volumetric interpolated examination before and after the administration of contrast medium, and diffusion weight imaging sequences in the axial plane with a calculation of the relative apparent diffusion coefficient (ADC) map. According to the mpMRI characteristics, a PIRADS score was assigned to each detected lesion.

### 2.3. Biopsy Technique

Our transperineal fusion biopsy protocol integrates both MRI-targeted and systematic sampling from the peripheral, anterior, and apical zones. The procedure, conducted under general or regional anesthesia with the patient in the lithotomy position, begins with the insertion of a bi-planar transrectal ultrasound (TRUS) probe (BK Medical, Peabody, MA, USA) mounted on a flexible arm (D&K Technologies GmbH, Barum, Germany). Using the BioJet system (D&K Technologies GmbH, Barum, Germany), the mpMRI and TRUS images are fused, allowing us to outline suspected lesions and the prostate’s contour on the mpMRI and overlay them on the TRUS image. Biopsies are then taken transperineally, guided by a 5 mm grid and a spring-loaded biopsy gun with an 18G needle. All procedures are performed by one of three urologists with extensive experience using the BioJet MRI/TRUS fusion biopsy system. For the peripheral zone, cores are taken following the extended 12-core scheme, while cores from the anterior zone and mid-anterior apex are obtained using the Ginsburg protocol [16,17]. Each biopsy core is subsequently assessed for the International Society of Urological Pathology (ISUP) grade group by a specialized genitourinary pathologist [18].

### 2.4. Study Outcomes

The study outcomes included both mpMRI findings and pathological results of the transperineal fusion biopsies. Primary outcomes were the PIRADS score and identification of CSPC from the targeted biopsies (targeted CSPC). Secondary outcomes were the number of suspicious lesions, any prostate cancer diagnosis, the identification of any clinically significant prostate cancer (overall CSPC), and insignificant prostate cancer results. In cases of multiple suspicious lesions on mpMRI, the higher PIRADS score was considered for analyses. CSPC was defined as ISUP ≥ 2, and the highest ISUP score was considered for calculations.

### 2.5. Statistical Analysis

Continuous variables were described as medians and interquartile ranges (IQRs) and categorical variables as numbers and percentages. Statistical comparisons between groups were performed by means of the chi-square, Fisher’s exact, and Mann–Whitney U tests. Univariate and multivariate logistic regressions were performed to calculate the association between 5-ARI treatment and outcomes. All analyses were 2-sided, and statistical significance was defined as *p* < 0.05. The statistical analyses were performed with SPSS v. 29 (IBM Corp., Armonk, NY, USA), and graphical items were created using RAWGraphs v. 2.0 (DensityDesign, Calibro and Inmagik, Milano, Italy) and SPSS v. 29.

## 3. Results

### 3.1. Study Population

After considering the inclusion and exclusion criteria, the cohort included 600 consecutive patients who had PIRADS ≥ 3 lesions on mpMRI and underwent combined transperineal fusion biopsy with a pathologic report. Fifty-two patients were treated with 5-ARIs for at least 6 months prior to mpMRI and 548 patients were not. Among the 5-ARI group, there were no patients treated by finasteride, only dutasteride. Table 1 presents the clinical and demographic characteristics of the study groups. 5-ARI patients were older with a median age of 72 years (IQR 69–75) compared to the median age of 68 years (IQR 63–73) of the others (*p* < 0.001), and they had more previous biopsies (69% vs. 47%, *p* = 0.004). The clinical risk stratification, including PSA levels and cT staging, were not different between the groups (*p* = 0.14 and *p* = 0.75, respectively). The median prostatic volume was higher among 5-ARI patients with a median of 60 mL (IQR 43–96) compared to the median of 54 mL (IQR 37–76) among the non-5-ARI group (*p* = 0.04).

### 3.2. mpMRI Suspicious Findings

**Figure 1:**
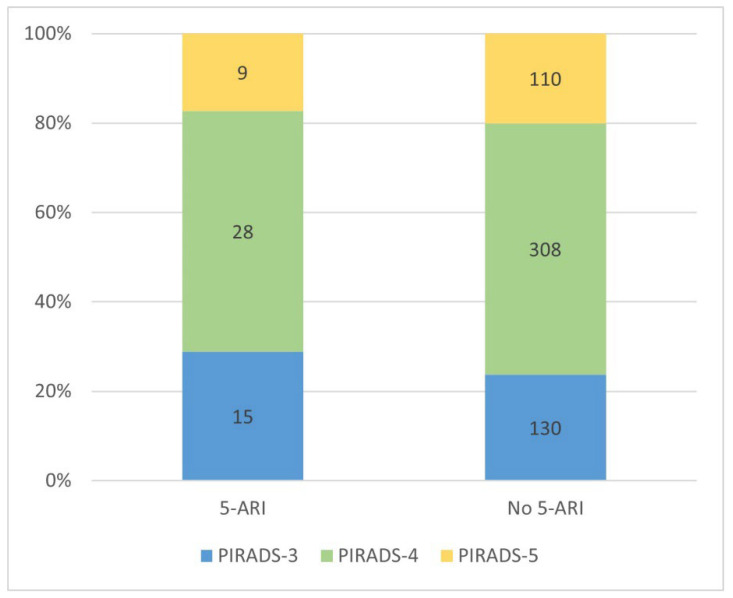
Comparison of PIRADS scores between 5-ARI groups. 5-ARI = 5-alpha reductase inhibitor; PIRADS = Prostate Imaging Reporting and Data System.

Most patients had multiple suspicious lesions (334 patients, 56%) and 266 (44%) had a solitary one. Overall, the prevalence of PIRADS scores of 3, 4, and 5 were 145 patients (24%), 336 patients (56%), and 119 patients (20%), respectively. PIRADS scores were 3, 4, and 5 in 15 (29%), 28 (54%), and 9 (17%) patients among the 5-ARI group and 130 (24%), 308 (56%), and 110 (20%) patients among the non-5-ARI group, and the scores were not different between the groups (*p* = 0.69, Figure 1). The number of suspicious lesions in mpMRI per patient were 1, 2, 3, and 4+ in 26 (50%), 20 (38%), 6 (12%), and 0 patients, respectively, among the 5-ARI group and 240 (44%), 209 (38%), 76 (14%), and 23 (4%) patients among the non-5-ARI group, respectively. There was no difference in the number of suspicious lesions per patient between the groups (*p* = 0.71). 5-ARI treatment was not associated with a PIRADS ≥ 4 score in the univariate logistic regression analysis (OR = 0.76, 95% CI 0.4–1.4, *p* = 0.41).

### 3.3. Biopsy Results

**Figure 2:**
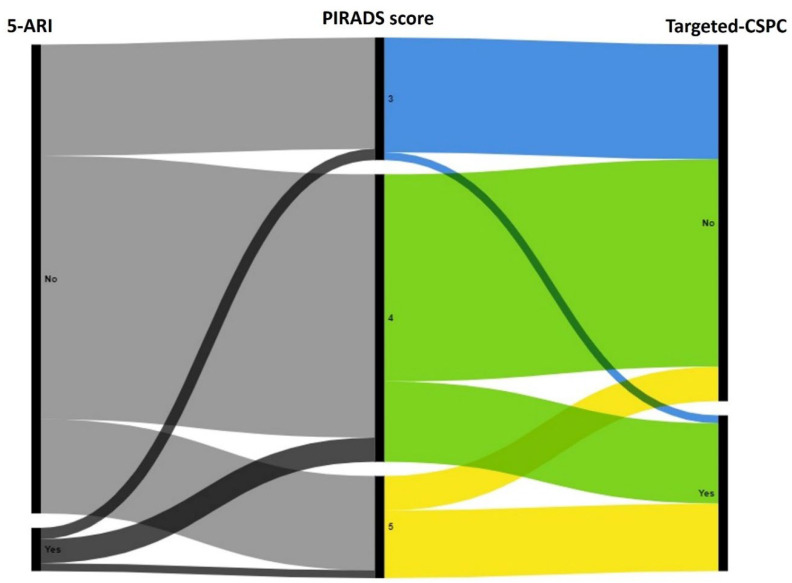
Primary outcomes of PIRADS scores on mpMRI and targeted CSPC detection rates after fusion biopsy among 5-ARI patients and non-5-ARI patients. 5-ARI = 5-alpha reductase inhibitor; CSPC = clinically significant prostate cancer; PIRADS = Prostate Imaging Reporting and Data System.

Any cancer, overall CSPC, targeted CSPC, and insignificant cancer were identified in 383 (63%), 211 (35%), 182 (30%), and 172 (29%) patients, respectively. The targeted CSPC detection rate among 5-ARI patients was 31% (16 patients), not different from the rate of 30% (166 patients) among the non-5-ARI group (*p* = 1). Rates of targeted CSPC for each PIRADS score were also not affected by 5-ARI treatment (Table 2). Secondary pathological outcomes of any cancer and overall CSPC were also not different between the groups (*p* = 0.45 and *p* = 0.17, respectively). The detection rate of insignificant prostate cancer was 30% among patients not treated with 5-ARI and higher compared to the rate of 13% in the 5-ARI group (*p* = 0.01, Table 2). 5-ARI treatment was not associated with targeted CSPC in the univariate logistic regression analysis (OR = 1.02, 95% CI 0.5–1.9, *p* = 0.94). Figure 2 illustrates a summary of the primary outcome results.

## 4. Discussion

In recent years, two pivotal studies, PROMIS (Prostate MR imaging study) and PRECISION (Prostate Evaluation for Clinically Important Disease: Sampling Using Image Guidance or Not?), have led to the emergence of the era of multiparametric magnetic resonance imaging (mpMRI) in the detection and management of prostate cancer (PCa) [4,19].

The PROMIS study assessed the accuracy of mpMRI-guided prostate biopsies compared to the standard transperineal template prostate mapping biopsies in men with suspected prostate cancer. In this prospective trial, 576 men underwent mpMRI followed by both types of biopsies. The study found that mpMRI had a sensitivity of 88% and a negative predictive value (NPV) of 76% for detecting ISUP grade 2 or higher cancer. The authors concluded that incorporating mpMRI into the diagnostic process could allow 27% of patients to avoid unnecessary biopsies for clinically significant prostate cancer [19].

Building on PROMIS, the PRECISION study evaluated an mpMRI-targeted diagnostic pathway versus a traditional TRUS-guided biopsy approach. This multicenter study included 500 men with suspected prostate cancer, with no biopsy performed for those with normal mpMRI results. In the standard-of-care arm, the detection rate for clinically significant cancer was 26%, while the rate for clinically insignificant cancer was 22%. Conversely, in the mpMRI-directed pathway, the detection rate for clinically significant cancer was 38%, with only 9% for clinically insignificant cancer. This mpMRI-directed approach led to a higher detection of clinically significant cancer and a reduction in the detection of clinically insignificant disease, all while requiring fewer biopsies and fewer cores sampled compared to the systematic TRUS-guided method [4].

Evaluating the studies mentioned above, the latest European Association of Urology (EAU) guidelines strongly recommend mpMRI for both biopsy-naive men and for men before repeat biopsies when the clinical suspicion of prostate cancer persists despite previous negative biopsies [20]. High-level evidence regarding the importance of mpMRI in the setting of prostate cancer screening and detection are accumulating. However, data are still lacking regarding several prevalent clinical scenarios. Symptomatic benign prostatic hyperplasia (BPH) is a highly prevalent condition among men at the age when prostate cancer screening is being considered. This results in many men with suspected prostate cancer receiving 5-ARIs used for alleviating voiding symptoms and preventing benign disease progression.

5-ARIs, including finasteride and dutasteride, are known to affect the cellular involution and epithelial shrinkage of benign prostatic tissue and increase the stromal/epithelial ratio in prostate cancer. It is suggested that 5-ARIs may induce significant phenotypic alterations in both BPH and prostate cancer [21]. In this regard, the use of 5-ARIs may affect the interpretation of mpMRI or the prostate biopsy results in 5-ARI-treated patients.

Our study was designed to evaluate the association between 5-ARI treatment of at least 6 months and alterations in the mpMRI-related diagnostic process of prostate cancer. We found that 5-ARI patients have a similar distribution of PIRADS scores for their mpMRI suspicious lesions compared to non-5-ARI patients and that the identification rates of CSPC, targeted or overall, are not affected by 5-ARIs. In addition, the rates of CSPC among each PIRADS score subgroup was also not related to 5-ARI treatment.

5-ARIs, such as finasteride and dutasteride, enhance hormonal alterations in the androgen pathway, resulting in a depletion of dihydrotestosterone, decrease in PSA, and a reduction in prostate size [5,22]. The association between 5-ARIs and prostate cancer has been already investigated before, concluding that although 5-ARIs reduce overall prostate cancer risk, it may increase the risk of high-grade disease in men who are undergoing regular screening for prostate cancer using PSA and rectal examination [23]. 5-ARIs were also suggested to serve as chemoprevention for disease progression during active surveillance [8,9]. All these data and conclusions were yielded in the pre-mpMRI era. mpMRI of the prostate has been identified as a test that may overcome some of diagnostic errors of prostate cancer, and emerging clinical trial data support the adoption of this technology as part of the standard of care for the diagnosis of prostate cancer [24]. Due to their hormonal effects, it seems reasonable that 5-ARIs might modify some of the mpMRI parameters, yet these possible modifications are unknown and insufficient data exist on how 5-ARI use affects mpMRI findings and the results of targeted fusion biopsies. Forte et al. demonstrated that the PIRADS score is sufficiently accurate in predicting prostate cancer among patients under 5-ARI treatment. Including PIRADS ≥ 3 lesions, sensitivity and negative predictive value were both 100%, with low specificity and low positive predictive value. Including PIRADS ≥ 4 lesions, the specificity and positive predictive value increased both to 79% and 80%, respectively [15].

Few prospective studies concluded that although dutasteride treatment of 6 months did not significantly influence the T2 contrast or the T2 relaxation values of mpMRI in men on active surveillance, it did increase the tumor apparent diffusion coefficient (ADC) and reduced conspicuity and tumor volume among them [11,12,13]. Giganti et al. found that following 6 months of dutasteride treatment, the mean ADC of the prostate was increased by 9% compared to −2% of the placebo group [12], suggesting that the lower threshold for targeted biopsies might be considered in men on dutasteride undergoing mpMRI. The MAPPED trial, a randomized, placebo controlled, double-blind clinical trial, assessed the effect of dutasteride on prostate cancer volume among men with low-risk disease on active surveillance. After 6 months of 5-ARI treatment, dutasteride was associated with a significant reduction in lesions’ volume on T2-weighted sequences compared to a placebo (36% vs. −12%, *p* < 0.001) [13]. Regarding PIRADS classification, similar to our findings, the retrospective study of Wang et al. reported no significant difference between the 5-ARI and non-5-ARI groups in the PIRADS score distribution, number of lesions, and lesion location [14]. However, as opposed to our results, their histologic findings indicated significantly fewer cancers among the 5-ARI group using saturation biopsies with or without targeted biopsies (46.3% vs. 68.0%; *p*  <  0.01). The multicenter Prostate MRI Outcome Database (PROMOD) study also included a comparison between 705 men receiving 5-ARI treatment for more than three months and 6913 5-ARI-naïve patients. The findings indicated that 5-ARI exposure did not influence the PIRADS distribution or the detection of CSPC. Although a statistically significant difference in serum PSA levels was observed, the levels in 5-ARI users were higher than anticipated [25].

Our results of no difference in mpMRI PIRADS scores and the targeted CSPC detection rate between 5-ARI patients and non-5-ARI patients suggest that the mpMRI-related diagnostic approach is appropriate for patients under 6 months of 5-ARI treatment. Our study is more updated, since it investigated PIRADS scores and targeted CSPC as primary outcomes and supplied a comparison to non-5-ARI patients, including subgrouping for each PIRADS score. The study of Forte et al. presented only data on diagnostic characteristics among 5-ARI patients, and although the ADC and tumor volume alterations [11,12] presented by Giganti et al. are indeed parameters for PIRADS score calculations, the final PIRADS score is the determinant for the decision of targeted biopsies. In addition, Wang et al. used saturation biopsies and reported the overall cancer detection rates instead of the targeted CSPC detection rates. We assume that the 5-ARI hormonal alterations in the androgen pathway does affect some of the mpMRI characteristics of the suspicious prostatic lesions; however, these are not enough to be translated into neither PIRADS score gradings nor targeted biopsy results.

In our study, a higher percentage of patients in the 5-ARI group had undergone a previous prostate biopsy compared to those not on 5-ARI (69% vs. 47%, *p* = 0.004). This may be attributed to the established evidence that 5-ARI use increases the sensitivity of detecting prostate tumors, particularly high-grade ones [26,27,28]. Consequently, the clinical threshold for recommending prostate biopsies in patients on 5-ARI treatment is lower, which could explain this finding. In a subgroup analysis limited to biopsy-naïve patients (5-ARI: *n* = 16; non-5-ARI: *n* = 292), although the difference in targeted CSPC rates was more pronounced (19% in the 5-ARI group vs. 37% in the non-5-ARI group), it remained statistically insignificant (*p* = 0.18). Future studies should be aware of this issue and address it appropriately.

This study has several limitations, mostly due to its retrospective nature. First, the mpMRI scans were performed by multiple institutes and interpreted by multiple radiologists, and a lack of standardization might expose the study to inter- and intra-observer variability bias, as reported in the literature [29]. Second, our results are patient-related and not lesion-related due to a lack of data. Third, 5-ARI treatment adherence of at least 6 months relied on patients’ statements and was not reassured objectively using electronic records of prescriptions. Despite these limitations, we believe that this study contributes clinically relevant findings and brings into attention an important topic scarcely mentioned in the literature.

## 5. Conclusions

PIRADS score assignments of suspicious lesions on mpMRI and targeted CSPC detection rates after transperineal fusion biopsies are not associated with 5-ARI treatment. A prostate cancer diagnostic approach using mpMRI is appropriate for patients under 5-ARI treatment, and they should not be addressed differently during this diagnostic process [30].

## Figures and Tables

**Table 1:** Comparison of baseline characteristics between 5-ARI groups. The continuous variables are reported as medians (IQR) and the categorical variables as numbers (%).

Characteristic	5-ARI (*n* = 52)	Non-5-ARI (*n* = 548)	*p* Value
Age (years)	72 (69–75)	68 (63–73)	**<0.001**
PSA (ng/dL)	6.8 (4–8.4)	6.6 (5–9.7)	0.14
Clinical T stage			0.75
T1c	38 (73%)	425 (78%)	
T2	13 (25%)	114 (21%)	
T3	1 (2%)	8 (1%)	
Family history	4 (8%)	79 (14%)	0.21
Previous biopsy	36 (69%)	256 (47%)	**0.004**
Active surveillance	15 (29%)	143 (26%)	0.74

5-ARI = 5-alpha reductase inhibitor; PSA = prostate-specific antigen. **Bold** indicates significant.

**Table 2:** Comparison of biopsy results between 5-ARI groups. The categorical variables are reported as numbers (%).

Characteristic	5-ARI (*n* = 52)	Non-5-ARI (*n* = 548)	*p* Value
Targeted CSPC	16 (31%)	166 (30%)	1
from PIRADS3	1 (6%)	8 (6%)	1
from PIRADS4	7 (25%)	87 (28%)	0.82
from PIRADS5	8 (89%)	71 (65%)	0.27
Any cancer	30 (57%)	353 (64%)	0.45
Overall CSPC	23 (44%)	188 (34%)	0.17
Insignificant cancer	7 (13%)	165 (30%)	**0.01**

5-ARI = 5-alpha reductase inhibitor; CSPC = clinically significant prostate cancer; PIRADS = Prostate Imaging Reporting and Data System. **Bold** indicates significant.

## Data Availability

The data that support the findings of this study are available upon request from the corresponding authors. The data are not publicly available due to ethical issues and the privacy of the participants.
